# P-1219. Broad spectrum of activity of AJ-099 against key refractory pathogens in cystic fibrosis lung infections: Pseudomonas aeruginosa and Mycobacterium abscessus

**DOI:** 10.1093/ofid/ofaf695.1412

**Published:** 2026-01-11

**Authors:** Sanghun Oh, Sungji Jung, Jiyoung Kim, Go-oun Kim, Dae Hun Kim, Su-Young Kim, Byung Woo Jhun, Hee-Jong Hwang, Si-Ho Kim

**Affiliations:** A&J BioLab Co., Ltd., Pohang, Kyongsang-bukto, Republic of Korea; A&J BioLab Co., Ltd., Pohang, Kyongsang-bukto, Republic of Korea; A&J BioLab Co., Ltd., Pohang, Kyongsang-bukto, Republic of Korea; A&J BioLab Co., Ltd., Pohang, Kyongsang-bukto, Republic of Korea; Samsung Medical Center, Sungkyunkwan University of School of Medicine, Seoul, Seoul-t'ukpyolsi, Republic of Korea; Samsung Medical Center, Sungkyunkwan University of School of Medicine, Seoul, Seoul-t'ukpyolsi, Republic of Korea; Samsung Medical Center, Sungkyunkwan University of School of Medicine, Seoul, Seoul-t'ukpyolsi, Republic of Korea; A&J Science, Daegu, Taegu-jikhalsi, Republic of Korea; Division of Infectious Diseases, Samsung Changwon Hospital, Sungkyunkwan University, Changwon, Kyongsang-namdo, Republic of Korea

## Abstract

**Background:**

Cystic Fibrosis (CF) is an autosomal recessive disorder caused by mutations in the cystic fibrosis transmembrane conductance regulator (CFTR) protein. Defects in the CFTR protein impair proper hydration of the cellular surface, leading to dysfunctional mucociliary clearance. These changes increase the risk of infections or co-infections by various bacterial pathogens, including *Pseudomonas aeruginosa*, non-tuberculous *Mycobacteria* (NTM) species. The rising prevalence of co-infections involving *P. aeruginosa*, *M. abscessus* in CF patients underscores the urgent need for novel therapeutic strategies.

**Figure:**
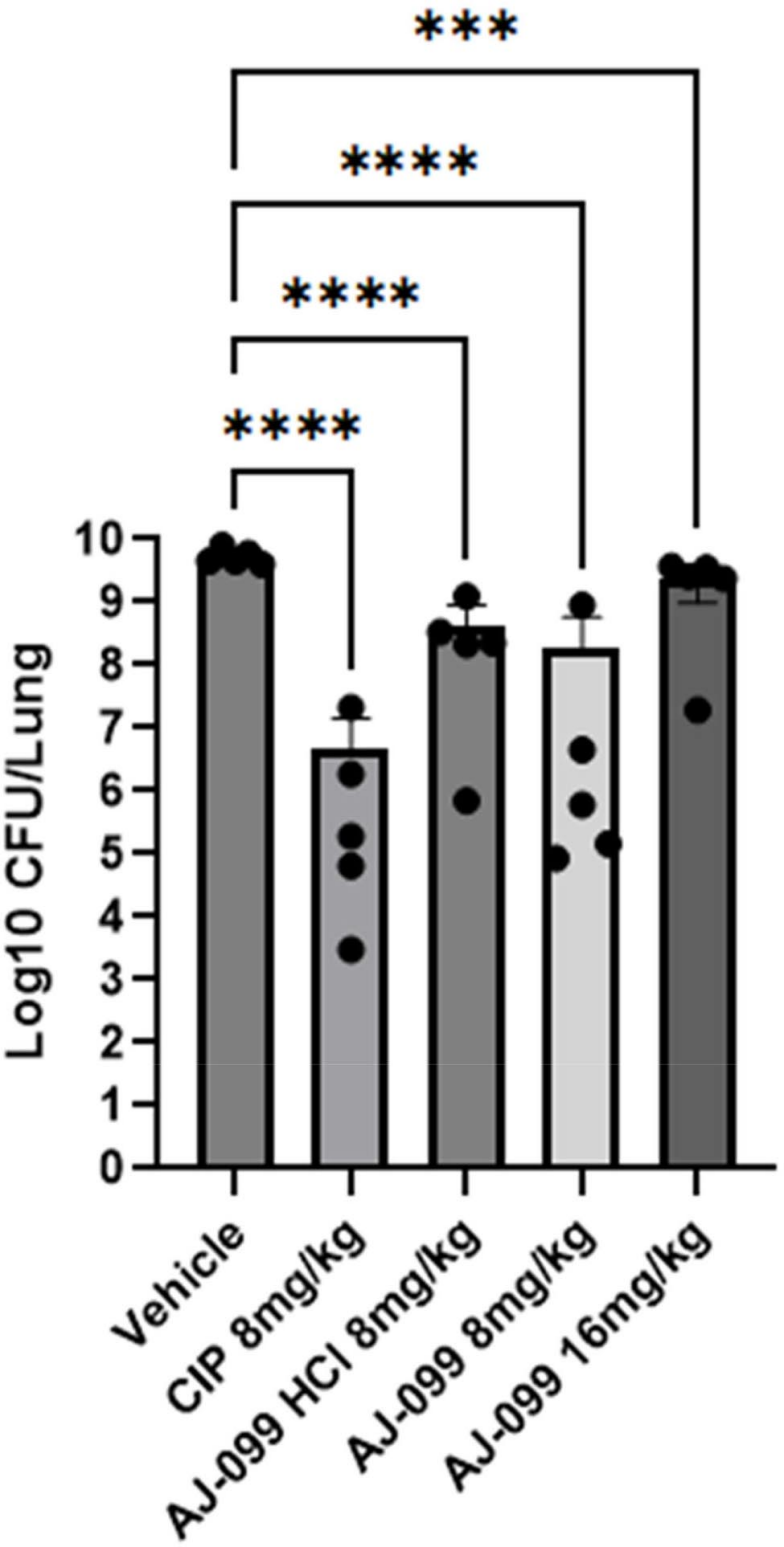
In vivo efficacy of AJ-099 against standard PAO1 P. aeruginosa

**Figure:**
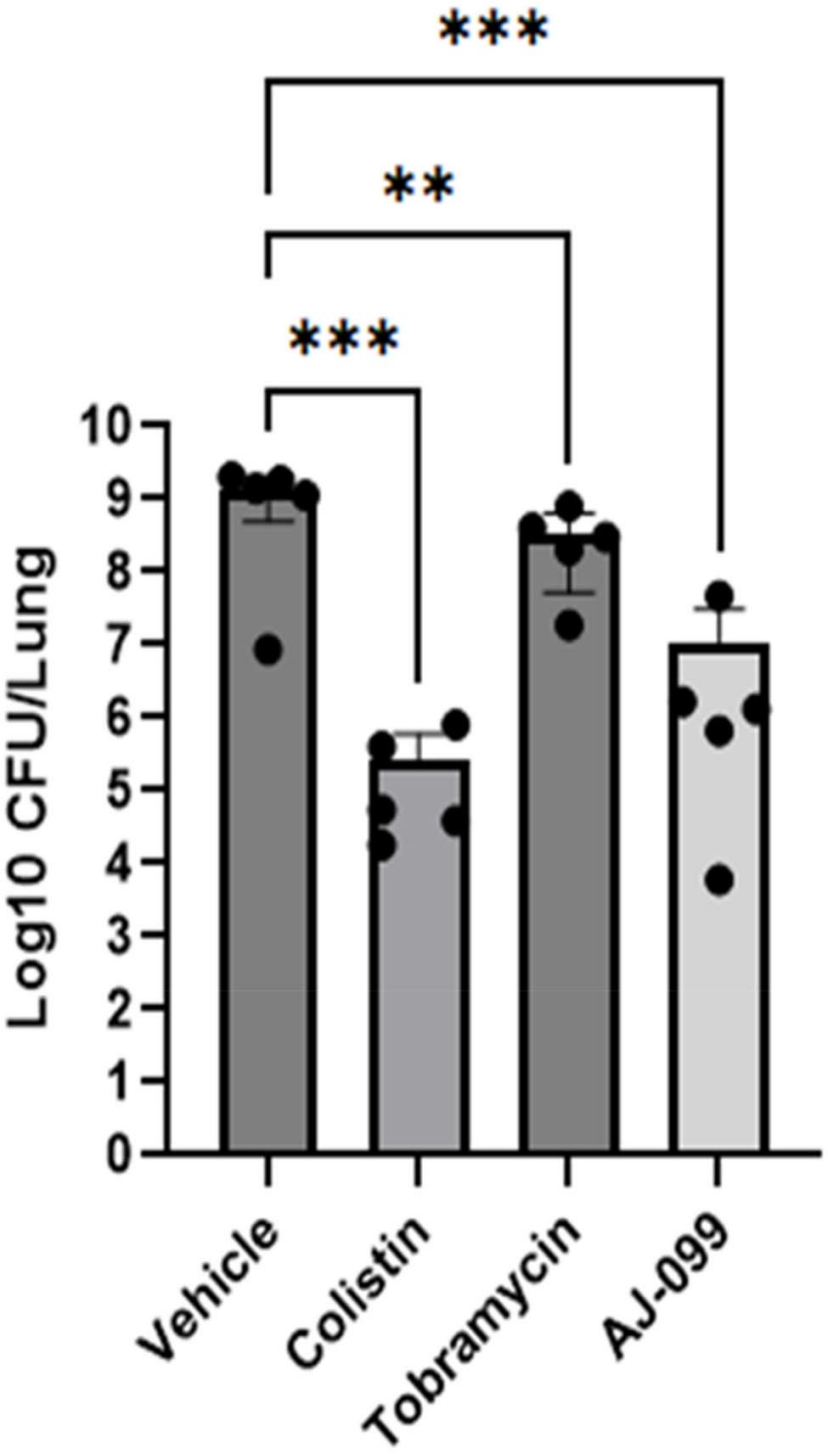
In vivo efficacy of AJ-099 against MDR P. aeruginosa

**Methods:**

Susceptibility testing of AJ-099 was conducted using the broth microdilution method against 78 *M. abscessus* clinical isolates from Samsung Medical Center in Seoul, Republic of Korea to determine its MIC_50_ and MIC_90_ values. Clarithromycin and amikacin resistant *M. abscessus* strains were included in the study.

The *in vivo* efficacy of AJ-099 free form and its HCl salt was evaluated in an ICR mouse model, in which mice were immunocompromised with cyclophosphamide and challenged with the standard *P. aeruginosa* PAO1 strain at an inoculation size of 5 x 10^6^ to 2 x 10^7^ CFU per mouse. The compounds were administrated via intranasal instillation at doses of 8 mg/kg or 16 mg/kg.

Additional *in vivo* studies were conducted using a non-immunocompromised ICR mouse model infected with a multidrug-resistant (MDR) clinical strain obtained from Samsung Medical Center in Changwon. Mice were challenged with *P. aeruginosa* (2.5×10^8^ to 1×10^9^ CFU per mouse), and the statistical significance of post-challenge changes in bacterial burden was assessed using one-way ANOVA.

**Figure:**
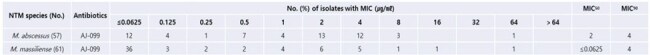
Antibacterial activity of AJ-099 against clinical isolates of M. abscessus

**Results:**

AJ-099 exhibits a potent antibacterial activity against various clinical *M. abscessus* strains, including those resistant to clarithromycin and amikacin. Its MIC_50_/_90_ values are 2 and 4 μg/mL, respectively.

In addition, treatment with AJ-099 in murine lung-infection models produced a statistically significant median reduction of 3.2 log₁₀ CFU in the pulmonary burden of multidrug-resistant *P. aeruginosa* (P < 0.005).

**Conclusion:**

Collectively, these findings support inhaled AJ-099 as a promising therapeutic candidate for treating refractory lung infections in CF patients.

**Disclosures:**

Sanghun Oh, n/a, A&J Science Co., Ltd.: Employee of A&J Science Sungji Jung, n/a, A&J Science Co., Ltd.: Employee of A&J Science Jiyoung Kim, n/a, A&J Science Co., Ltd.: Employee of A&J Science Go-oun Kim, n/a, A&J Science Co., Ltd.: Employee of A&J Science Hee-Jong Hwang, PhD, A&J Science Co., Ltd.: Board Member|A&J Science Co., Ltd.: Ownership Interest

